# Development and validation of open-source software for DNA mixture interpretation based on a quantitative continuous model

**DOI:** 10.1371/journal.pone.0188183

**Published:** 2017-11-17

**Authors:** Sho Manabe, Chie Morimoto, Yuya Hamano, Shuntaro Fujimoto, Keiji Tamaki

**Affiliations:** 1 Department of Forensic Medicine, Kyoto University Graduate School of Medicine, Kyoto, Japan; 2 Forensic Science Laboratory, Kyoto Prefectural Police Headquarters, Kyoto, Japan; Universitat Pompeu Fabra, SPAIN

## Abstract

In criminal investigations, forensic scientists need to evaluate DNA mixtures. The estimation of the number of contributors and evaluation of the contribution of a person of interest (POI) from these samples are challenging. In this study, we developed a new open-source software “*Kongoh*” for interpreting DNA mixture based on a quantitative continuous model. The model uses quantitative information of peak heights in the DNA profile and considers the effect of artifacts and allelic drop-out. By using this software, the likelihoods of 1–4 persons’ contributions are calculated, and the most optimal number of contributors is automatically determined; this differs from other open-source software. Therefore, we can eliminate the need to manually determine the number of contributors before the analysis. *Kongoh* also considers allele- or locus-specific effects of biological parameters based on the experimental data. We then validated *Kongoh* by calculating the likelihood ratio (LR) of a POI’s contribution in true contributors and non-contributors by using 2–4 person mixtures analyzed through a 15 short tandem repeat typing system. Most LR values obtained from *Kongoh* during true-contributor testing strongly supported the POI’s contribution even for small amounts or degraded DNA samples. *Kongoh* correctly rejected a false hypothesis in the non-contributor testing, generated reproducible LR values, and demonstrated higher accuracy of the estimated number of contributors than another software based on the quantitative continuous model. Therefore, *Kongoh* is useful in accurately interpreting DNA evidence like mixtures and small amounts or degraded DNA samples.

## Introduction

In forensic casework, the DNA typing system for short tandem repeat (STR) loci is used for criminal investigations. STR alleles of approximately 15 loci are amplified simultaneously in the polymerase chain reaction (PCR) process, and the repeated numbers are determined through the observed allelic peaks in capillary electrophoresis. As commercially available kits are highly sensitive, the correct genotype is usually identified using less than 1 ng of human genomic DNA. The likelihood ratio (LR) is often used for interpreting DNA evidence by calculating the weight of the evidence from the ratio of likelihood of a prosecution hypothesis (e.g., a suspect is a contributor) and that of a defense hypothesis (e.g., a suspect is not a contributor). If LR > 1, the evidence supports the prosecution hypothesis; however, if LR < 1, the evidence supports the defense hypothesis. However, forensic scientists often need to evaluate DNA mixtures of two or more individuals in criminal investigations. The estimation of the number of contributors and evaluation of the contribution of a person of interest (POI), such as victim or suspect, by using these samples is very challenging. The obtaining of complete profiles from these samples may not be feasible because some alleles might not be recorded if the peak they generate is below a threshold (i.e., allelic drop-out). Small allelic peaks derived from the minor contributors in a DNA sample may not be distinguished from artifacts called −1 backward stutter peaks (i.e., one repeat shorter than the allele), which result from the slippage product of the STR allele in the PCR process [1].

The three types of LR-based interpretational models for mixtures and small amount of DNA samples are binary, semi-continuous (qualitative continuous), and fully continuous (quantitative continuous). The conventional method of interpreting DNA mixture is based on a binary model, wherein the probability of the evidence is assigned as zero or one [2]. In general, this model does not use peak height information (i.e., unrestricted combinatorial approach [2]), and does not consider allelic drop-out; therefore, it cannot be employed for interpreting small amounts of DNA profiles. The qualitative continuous model allows for improved interpretation of these profiles by using the drop-out probability [3]. Although the open-source software based on this model has been developed (e.g., *LRmix Studio* [4] and *Lab Retriever* [5]), this model does not use peak height information, and the user must call peaks as alleles and assign stutter peaks. Therefore, stutter peaks can be assigned as allelic and accounted for as an extra contributor.

Some countries have begun using a quantitative continuous model to calculate rigorous LR values for investigating whether a POI is a contributor in a crime stain profile. In this model, the peak height information is used and allelic drop-outs are considered for calculating the probability of obtaining a profile, given all possible genotype combinations of the contributors. Therefore, a fixed filter for removing stutter peaks does not require to be used for determining whether a peak in the stutter position represents a stutter or an allele. Thus, this model can avoid the subjectivity induced by forensic scientists [6]. Software have been developed based on the quantitative continuous models: *DNA·VIEW Mixture Solution*^*TM*^, *LiRaHT* [7], *DNAmixtures* [8], *EuroForMix* [9], and *likeLTD* [10] use the gamma distribution to model peak heights, while *CEESIt* [11] uses normal distribution. Furthermore, *STRmix* [12] and *TrueAllele* [13] perform Markov chain Monte Carlo simulation to estimate the distributions of peak heights by incorporating many biological parameters.

However, there are some problems in these current software based on the quantitative continuous model. All software based on the model are commercial or proprietary, except for *EuroForMix*, *likeLTD*, and *CEESIt*. The lack of availability of software has inhibited comparative studies. In open-source software *likeLTD* and *CEESIt*, we can hypothesize only up to three contributors in the mixture. Furthermore, the number of contributors must be determined manually before the software analysis in all three open source software.

In the current study, we developed a new open-source software called *Kongoh* (the Japanese word for “mixture”) based on a quantitative continuous model. The software consists of a graphical user interface written in R language (version 3.3.2) [14] and the source code is freely available at GitHub (https://github.com/manabe0322/Kongoh/releases). Unlike other open-source software, likelihood values in both hypotheses of up to four contributor(s) are automatically calculated, and the number that shows maximum likelihood is regarded as the best number. Therefore, the number of contributors does not need to be determined manually before software analysis. In *Kongoh*, LR is calculated as a ratio of the maximum likelihood in the prosecution hypothesis and that in the defense hypothesis. Furthermore, likelihood calculation is based on the biological parameters determined through experimental data to interpret DNA mixture accurately.

In this study, we validated the software by referring to the guidelines for the validation of probabilistic genotyping systems published by the Scientific Working Group on DNA Analysis Methods (SWGDAM) [15]. We performed the validations based on the following metrics: sensitivity (i.e., LR of true contributors), specificity (i.e., LR of non-contributors), precision (i.e., reproducibility of LR values), accuracy of calculations, and case-type samples. Calculations in *Kongoh* were compared with those obtained from a binary model, a qualitative continuous model (*LRmix Studio*), and another quantitative continuous model (*EuroForMix*) and the differences or the similarities of the results were checked. We also investigated the accuracy of the estimated number of contributors in each model.

## Methods

### DNA profiles used in *Kongoh*

The profile of 15 STR loci typed by the Identifiler^®^ Plus system can be interpreted using the current version of *Kongoh*. The system is run for 28 amplification cycles. The PCR products are analyzed using an ABI 3130xl Genetic Analyzer with 10-s injection time, and the data are analyzed using GeneMapper^TM^ Software (Thermo Fisher Scientific). We used 30 relative fluorescence units (RFU) as the analytical threshold (AT) for peak detection. The peak located at the position of the −1 backward stutter does not need to be designated as an allele or stutter because the derivation of the peak can be determined probabilistically by using *Kongoh*. Forward stutters and −2 backward stutters are not considered in the current version of *Kongoh* because these peaks are considerably small and not relevant for mixture interpretation [16]. *Kongoh* also considers allelic drop-out, as explained in the S1 Appendix. Allelic drop-in, which refers to small unexpected peaks, does not need to be considered in 28 cycles because drop-in tends to be observed for higher amplification cycles [17]. If drop-in peaks are observed, they are explained by additional unknown contributors in *Kongoh* [8].

### LRs

The LR compares the conditional probabilities of obtaining the crime stain profile (*CSP*) under the prosecution hypothesis (*H*_*p*_) and defense hypothesis (*H*_*d*_). A POI is assumed present under *H*_*p*_ but not under *H*_*d*_. The LR is defined as follows:

$$\mathrm{LR}=\frac{f(CSP|H_{p})}{f(CSP|H_{d})}$$ (1)

If LR > 1, the evidence supports *H*_*p*_, but if LR < 1, the evidence supports *H*_*d*_.

For DNA mixture interpretation based on the quantitative continuous model in *Kongoh*, the numerator and denominator of the right-hand side term in Eq (1) are calculated as follows:

$$f\left(CSP|H\right)=\prod_{l}\sum_{i}f\left(CSP_{l}|G_{l,i}\right)\mathrm{Pr}(G_{l,i}|H)$$ (2)

where *f*(*CSP*_*l*_|*G*_*l*,*i*_) is the probability density of obtaining the *CSP* given an *i*th (*i* = 1, 2,…*I*) genotype combination *G*_*l*,*i*_ of all contributors in locus *l*. *f*(*CSP*_*l*_|*G*_*l*,*i*_) is called weight (*w*_*l*,*i*_), which represents a goodness of fit of the observed peak heights to the genotype combination *G*_*l*,*I*_ [12]. Pr(*G*_*l*,*i*_*|H*) is the probability of obtaining *G*_*l*,*i*_ given a hypothesis *H*, which represents the contributor combination (e.g., POI and two unknowns contribute to *CSP*). In other words, Pr(*G*_*l*,*i*_*|H*) represents the frequency of the genotype combination *G*_*l*,*i*_. The sub-population effect can be considered for calculating the genotype frequency in *Kongoh*. *G*_*l*,*i*_ sets differ as per the number of contributors hypothesized in *H*.

Eq (1) for the LR is rewritten as follows:

$$\mathrm{LR}=\frac{\prod_{l}\sum_{i}w_{l,i}\mathrm{Pr}(G_{l,i}|H_{p})}{\prod_{l}\sum_{i^{\prime }}w_{l,i^{\prime }}\mathrm{Pr}(G_{l,i^{\prime }}|H_{d})}$$ (3)

where G_*l*,(1,2,…,*I*)_ are specified by *H*_*p*_ and G_*l*,(1,2,…,*I'*)_ are specified by *H*_*d*_. In the binary model, the *w*_*l*,*i*_ or *w*_*l*,*i'*_ values are assigned zero or one depending on whether the evidence is deemed impossible or possible if it originated from the specified genotype combinations. In the quantitative continuous model, the *w*_*l*,*i*_ or *w*_*l*,*i'*_ values are determined continuously by comparing the observed peak heights with the expected peak heights calculated using biological parameters.

These equations have also been used in other software based on the quantitative continuous model [12]; however, we changed some parameters and the computational principle. In *Kongoh*, the *w*_*l*,*i*_ or *w*_*l*,*i'*_ values are determined using the following five biological parameters: mixture ratio (*MR*_*n*_), DNA degradation (*d*), locus-specific amplification efficiency (*AE*_*l*_), heterozygote balance (*Hb*_*al*_), and stutter ratio (*SR*_*al*_). The subscripts of each parameter denote the contributor (*n*), allele (*a*), or locus (*l*). The variance of these parameters was considered according to the peak height values. To estimate the distributions of the expected peak heights, a Monte Carlo simulation is performed based on the probability distributions of the five parameters determined through the experimental data [18, 19]. The peak heights generated through the simulation are then approximated using gamma distributions.

**Fig 1:**
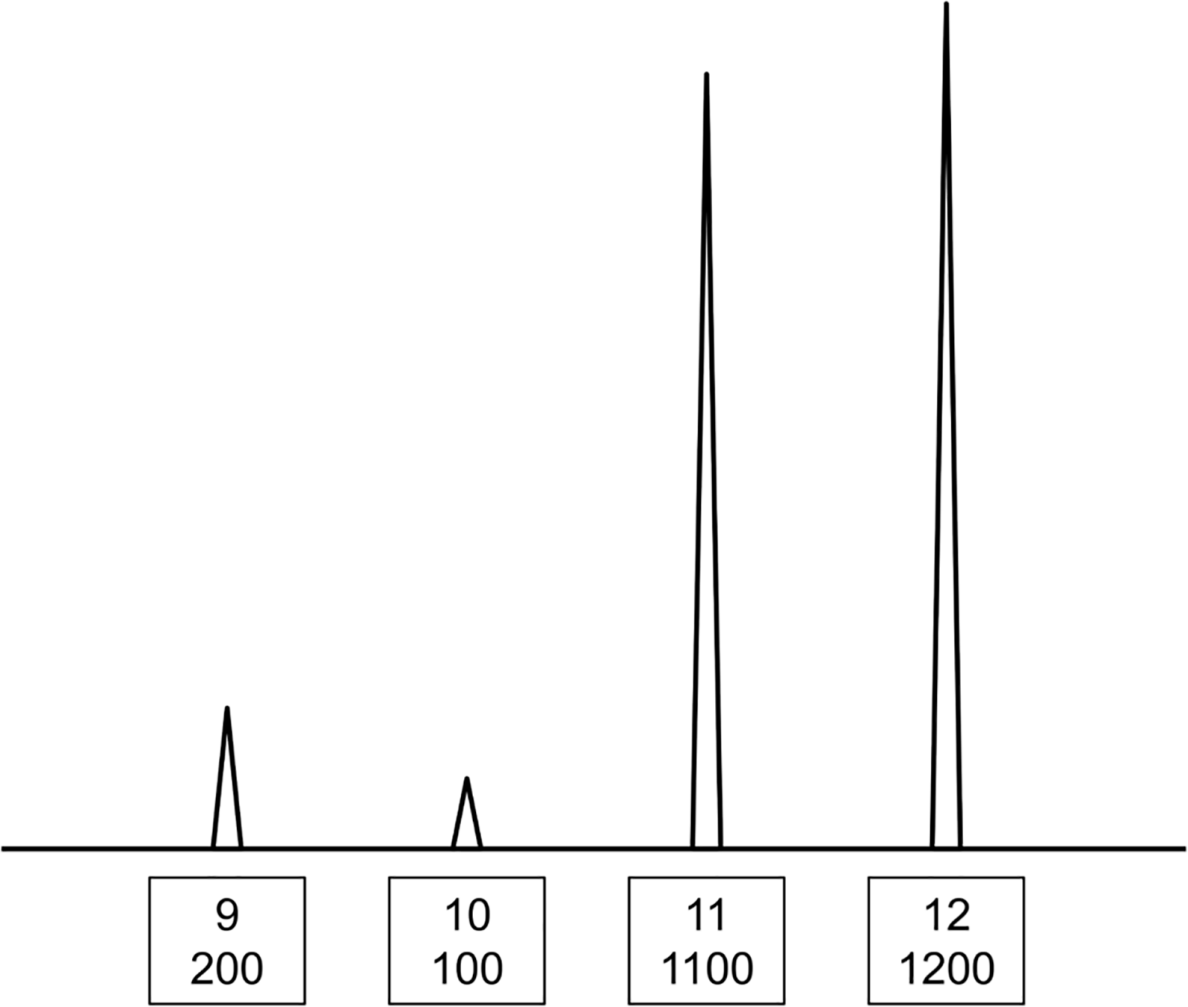
Example of the crime stain profile (*CSP*) in a single locus.

The likelihoods of 1–4 contributors in both *H*_*p*_ and *H*_*d*_ are calculated according to Eq (2), and the number that shows maximum likelihood is regarded as best (i.e., the best *H*). The likelihoods of any number of contributors can be theoretically calculated; however, there is a practical computational time limit that restricts *Kongoh* to a maximum of four contributors. *MR*_*n*_ and *d* are also estimated based on the maximum likelihood in both *H*_*p*_ and *H*_*d*_. In *Kongoh*, LR is calculated as the ratio of the maximum likelihood in *H*_*p*_ to that in *H*_*d*_; therefore, the hypothesized numbers of contributors may be different in *H*_*p*_ and *H*_*d*_. For example, if a POI’s genotype is (10, 11) in the mixed profile shown in Fig 1, explaining the mixture as a two-person contribution in *H*_*p*_ will be difficult because of the heterozygous peak imbalance of the POI and the other contributor. Thus, three or more contributors are required in *H*_*p*_. On the other hand, the mixture can be easily explained as two contributors in *H*_*d*_ by considering the genotype combination of (9, 10) and (11, 12).

**Fig 2:**
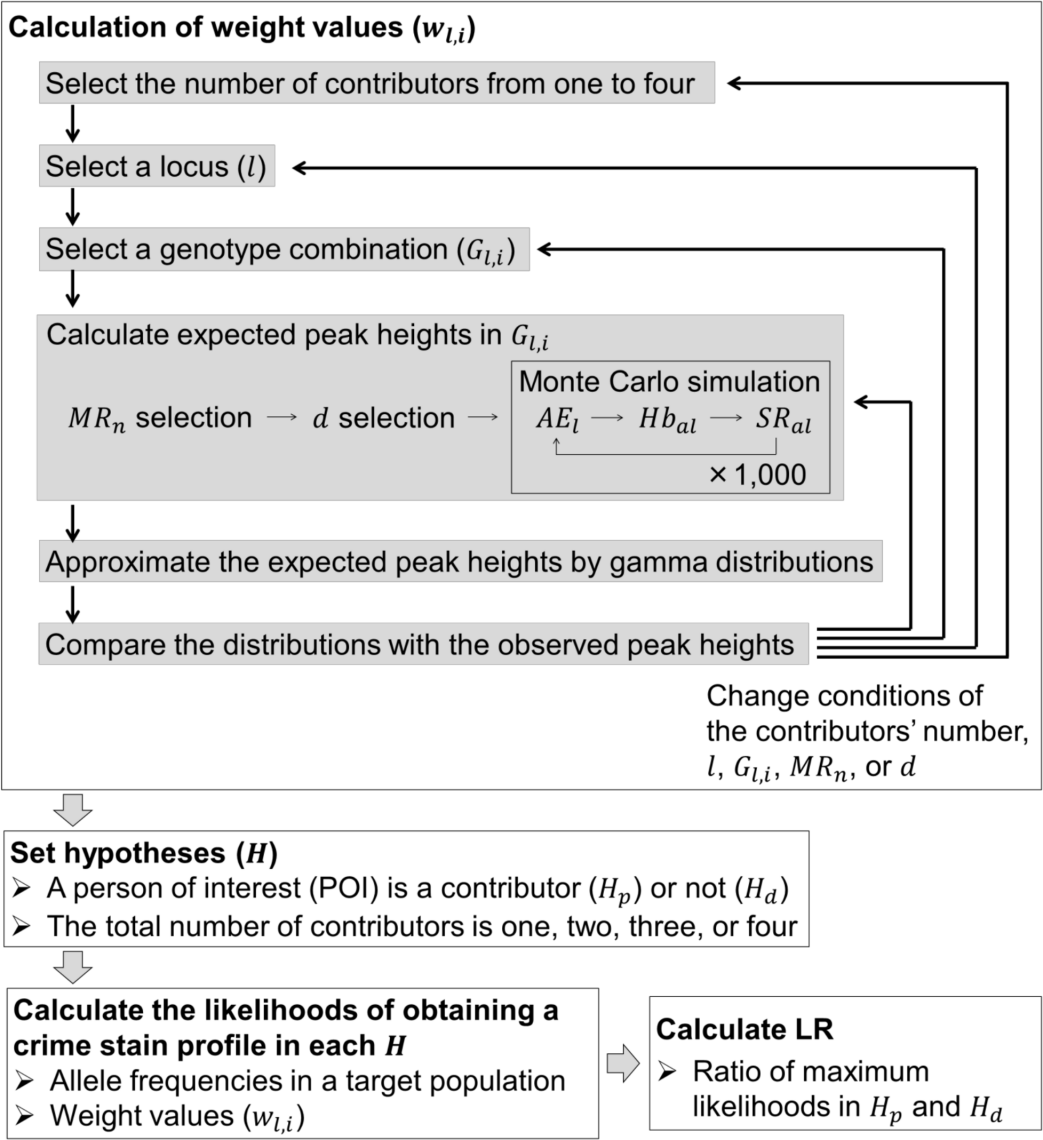
Procedure for calculating LR in *Kongoh*.

Fig 2 shows the procedure for calculating the LR in Eq (3). First, we calculated *w*_*l*,*i*_ of all *G*_*l*,*i*_ for 1–4 contributors. Then, we set hypotheses (*H*), which represent the contributor combinations. We investigate both *H*_*p*_ and *H*_*d*_ by changing the number of contributors from one to four. We can incorporate other known profiles (e.g., a victim’s profile) in both *H*_*p*_ and *H*_*d*_. Next, we calculate the likelihoods of obtaining *CSP* in all *H* using allele frequencies in a target population and *w*_*l*,*i*_. After the likelihoods of all *H* (i.e., both *H*_*p*_ and *H*_*d*_ in 1–4 contributors) are calculated, an LR value is calculated using the ratio of the maximum likelihood in *H*_*p*_ to that in *H*_*d*_. We have provided a detailed explanation of the procedure in the S1 Appendix.

### Testing set

*Kongoh* was validated using 27 two-person mixtures, 27 three-person mixtures, and 18 four-person mixtures. These mixtures were experimentally prepared using non-degraded DNA from pristine blood samples. We created three sets of two-person mixtures, in which the DNA samples of each pair were mixed in ratios of 1:1, 3:1, and 9:1. Further, we created three sets of three-person mixtures, in which the DNA samples of each trio were mixed in ratios of 1:1:1, 3:2:1, and 8:1:1. Finally, we created two sets of four-person mixtures, in which the DNA samples of each quartet were mixed in ratios of 1:1:1:1, 4:3:2:1, and 7:1:1:1. We chose the contributors’ combinations in some mixtures to have many masked/shared alleles. For example, the maximum number of alleles was four in one set of three- and four-person mixture each; therefore, these mixtures could be considered as two-person contributions (S1 Fig). The total DNA concentration of each mixture was adjusted to 0.1, 0.05, and 0.025 ng/μl, and 10 μl DNA solutions of all the mixtures were analyzed according to the description in “DNA profiles used in *Kongoh*”.

All blood donors signed written consent forms prior to donation. This study was approved by the ethics committee of Kyoto University Graduate School and Faculty of Medicine (number G1051) and conducted according to the guidelines approved by that committee. The committee has placed data sharing restrictions to protect personal information. Therefore, with respect to the profiles of DNA mixtures and the genotypes of each individual, the sample approval allowed us to use these data for only this study. The datasets generated and/or analyzed during the current study are not publicly available owing to participant confidentiality, but will be available from the corresponding author on reasonable request after obtaining ethical approvals from the ethics committee of Kyoto University Graduate School and Faculty of Medicine (http://www.ec.med.kyoto-u.ac.jp/index.html).

We also investigated 4 two-person profiles through DNA degradation by UV exposure and 4 two-person profiles with PCR inhibition by using humic acid, which were obtained from the publicly available data sets in Project Research Openness for Validation with Empirical Data (PROVEDIt: http://sites.bu.edu/grgicak/provedit/). These DNA samples were already amplified using the Identifiler Plus kit with 28 cycles, and analyzed using an ABI 3130xl Genetic Analyzer with 10-s injection time.

### Validation by referring to the SWGDAM guidelines

We validated *Kongoh* by referring to the SWGDAM guidelines [15, 20]. Developmental validation of the SWGDAM guidelines includes conceptual validation [21], that is, publishing the underlying scientific principle(s) of the software in a peer-reviewed scientific journal. The underlying principles and characteristics of *Kongoh* have been discussed in “Likelihood ratios,” and a detailed explanation is provided in the S1 Appendix.

Developmental validation also includes operational validation [21], that is sensitivity, specificity, precision, accuracy, and case-type samples. For calculating LR in sensitivity validation, we designated two POIs in each mixture. One POI was the contributor with the largest amount of DNA, and the other was the contributor with the smallest amount of DNA. For example, in a mixture with person A:person B:person C = 3:2:1, we first designated person A as the major POI and persons B and C as unknown contributors. Second, we designated person C as the minor POI and persons A and B as unknown contributors. We also selected person A or C as POI and remaining persons as unknown contributors in the 1:1:1 mixture. Then, we calculated two LR values for the major and minor POIs in each mixture. In specificity validation, we computationally generated genotypes of 100 non-contributors based on Japanese allele frequencies [22], and then calculated the LR assuming each non-contributor as a POI in six mixtures each of two-, three-, and four-persons. The precision validation was performed by repeating the LR calculation of true contributors and non-contributors. We then investigated the correlation of LR values between each run through Pearson’s product-moment correlation test using R language [14]. *P* < 0.05 was considered statistically significant. For accuracy validation, we confirmed the accuracy of each calculation performed using the *Kongoh* program.

The testing set included case-type samples of stutter, masked/shared alleles, differential and preferential amplifications, degradation, and inhibition. The following mixtures were the samples in which distinguishing minor allelic peaks from stutters was difficult: 9:1, 8:1:1, and 7:1:1:1. Four-person mixtures or samples with different DNA amount between contributors (e.g., 9:1 mixtures) had some masked/shared alleles. In particular, one set each of three- and four-person mixtures could be explained as two contributors because of many masked/shared alleles. Differential amplification is defined as the selection of one locus over another during the PCR [23]. Preferential amplification is defined as the unequal sampling of two heterozygote alleles present in a locus due to stochastic fluctuation arising when only a few DNA molecules are used to initiate the PCR [23]. Differential and preferential amplifications could potentially occur in small amounts of DNA samples, mixtures with minor contributors, and DNA degradation. We used profiles with DNA degradation and those with PCR inhibition obtained from the PROVEDIt dataset. To calculate genotype frequencies in the likelihoods, Japanese allele frequencies [22] were used for our experimental dataset, and Caucasian allele frequencies [24] were used for the PROVEDIt dataset.

### Comparative studies with other models

The LR values generated from true-contributor testing were compared with those of a binary model, *LRmix Studio* (version 2.1.3), and *EuroForMix* (version 1.7). We used the same 6 two-person, 6 three-person, and 6 four-person mixtures as those used in non-contributor testing. The LR values of all models were also calculated according to the ratio of maximum likelihoods in *H*_*p*_ and *H*_*d*_ assuming a 1–4 person contribution.

For the binary model, all the mixtures were genotyped using 150 RFU as the AT and −1 backward stutters were removed by the stutter filters in each locus. In general, 150 RFU corresponds to the threshold for avoiding the interpretation of DNA profiles with allelic drop-out [25]; therefore, we used the threshold as a substitute for AT in the binary model. The likelihoods were calculated using *LRmix Studio* by setting the drop-out probability Pr(D) = 0, the drop-in probability Pr(C) = 0, and the theta correction Fst = 0 (i.e., without considering sub-population effect in the validation).

In *LRmix Studio*, Pr(D) was determined by the maximum likelihood estimation in each hypothesis of 1–4 contributors [26]. We set Pr(C) = 0 and Fst = 0, which is the same condition as that in the *Kongoh* analysis. All the mixtures were genotyped using 30 RFU as the AT and −1 backward stutters were removed using the stutter filters.

In *EuroForMix*, we again set Pr(C) = 0 and Fst = 0. We considered the effect of degradation and stutter. All mixtures were genotyped using 30 RFU as the AT and the stutter filters were removed.

### Determination of the number of contributors

We confirmed the accuracy of the estimated number of contributors in each mixture. The confirmation was performed by comparing the likelihoods of 1–4 unknown contributors, and the number showing the maximum likelihood was determined as the number of contributors. We then compared the results with those of the binary model, *LRmixStudio*, and *EuroForMix* using the same 6 two-person, 6 three-person, and 6 four-person mixtures as those used in non-contributor testing.

## Results

### LRs in true-contributor testing

**Fig 3:**
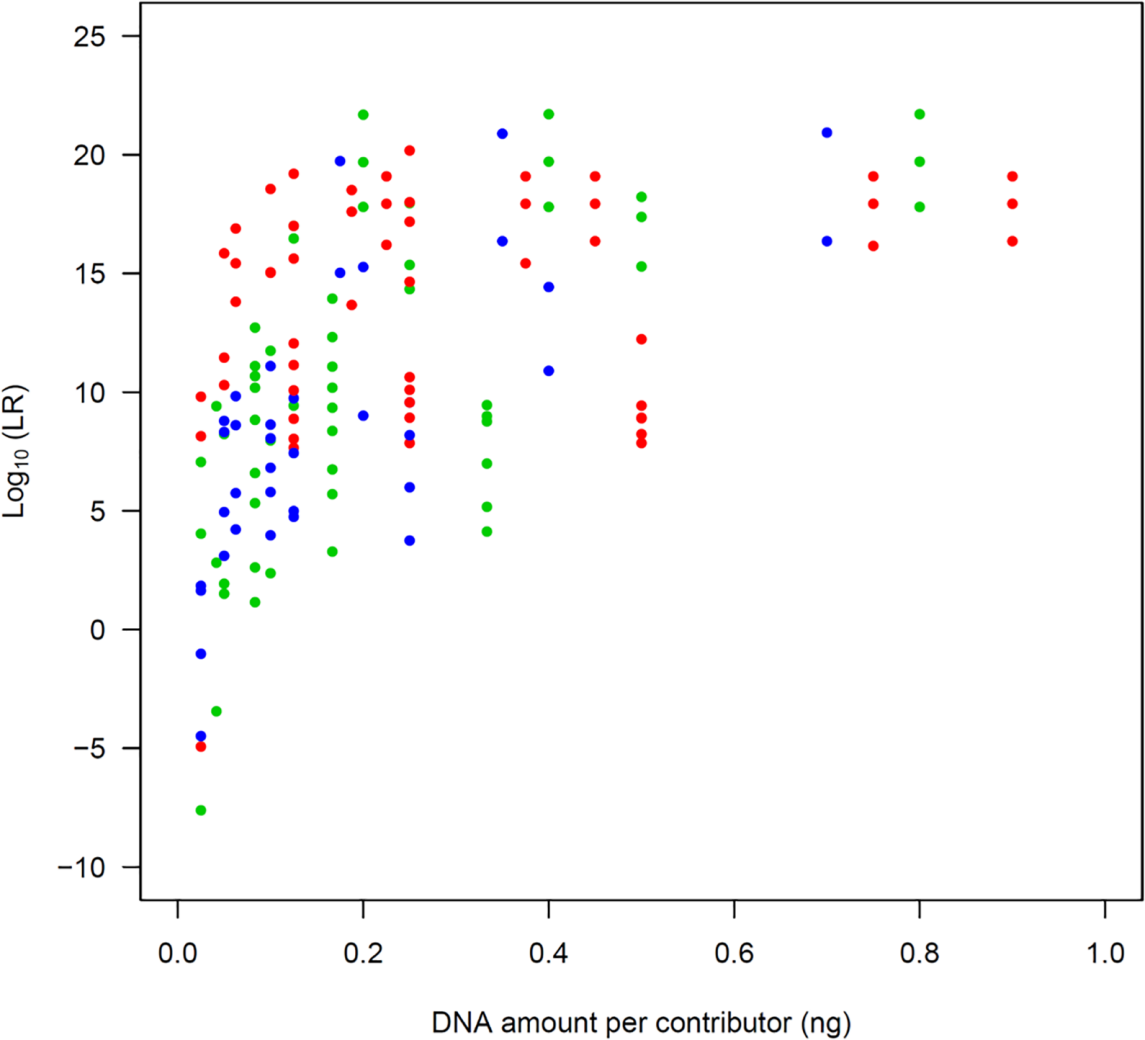
Plots of POI’s DNA amount vs LR values in true-contributor testing. The red, green, and blue plots represent LR values of two-person, three-person, and four-person mixtures, respectively.

We validated sensitivity using profiles of 27 two-person mixtures, 27 three-person mixtures, and 18 four-person mixtures, including samples of various DNA amounts and mixture ratios, without degradation and inhibition. We considered a true major contributor and a true minor contributor as POIs in each mixture, and calculated the LR values of each POI (Fig 3). The LR values tended to decrease as the amount of DNA of the POI decreased. The LR values of the POI with more than 0.1 ng tended to be greater than 10,000, which can be considered as a “very strong evidence to support” the contribution by the POI [27]. Even in the case-type samples, wherein it is difficult to distinguish small allelic peaks from stutter peaks, and with allelic products of minor contributor(s) masked by large allelic peaks, the LR values in true-contributor testing showed high sensitivity. For example, in a 4-person mixture with many masked/shared alleles (S1 Fig), the LR values for the major and minor POIs were 2.27 × 10^16^ and 4.47 × 10^8^, respectively. In mixtures with allelic drop-outs due to small amounts of DNA, LR values were greater than one if the number of drop-outs was less than four. However, there were five mixtures that exhibited LR < 1 (i.e., Type I errors). The DNA amount of the POI in these five mixtures was only 25 or 42 pg, and 5–11 drop-outs of POI alleles were confirmed.

### LRs in non-contributor testing

**Fig 4:**
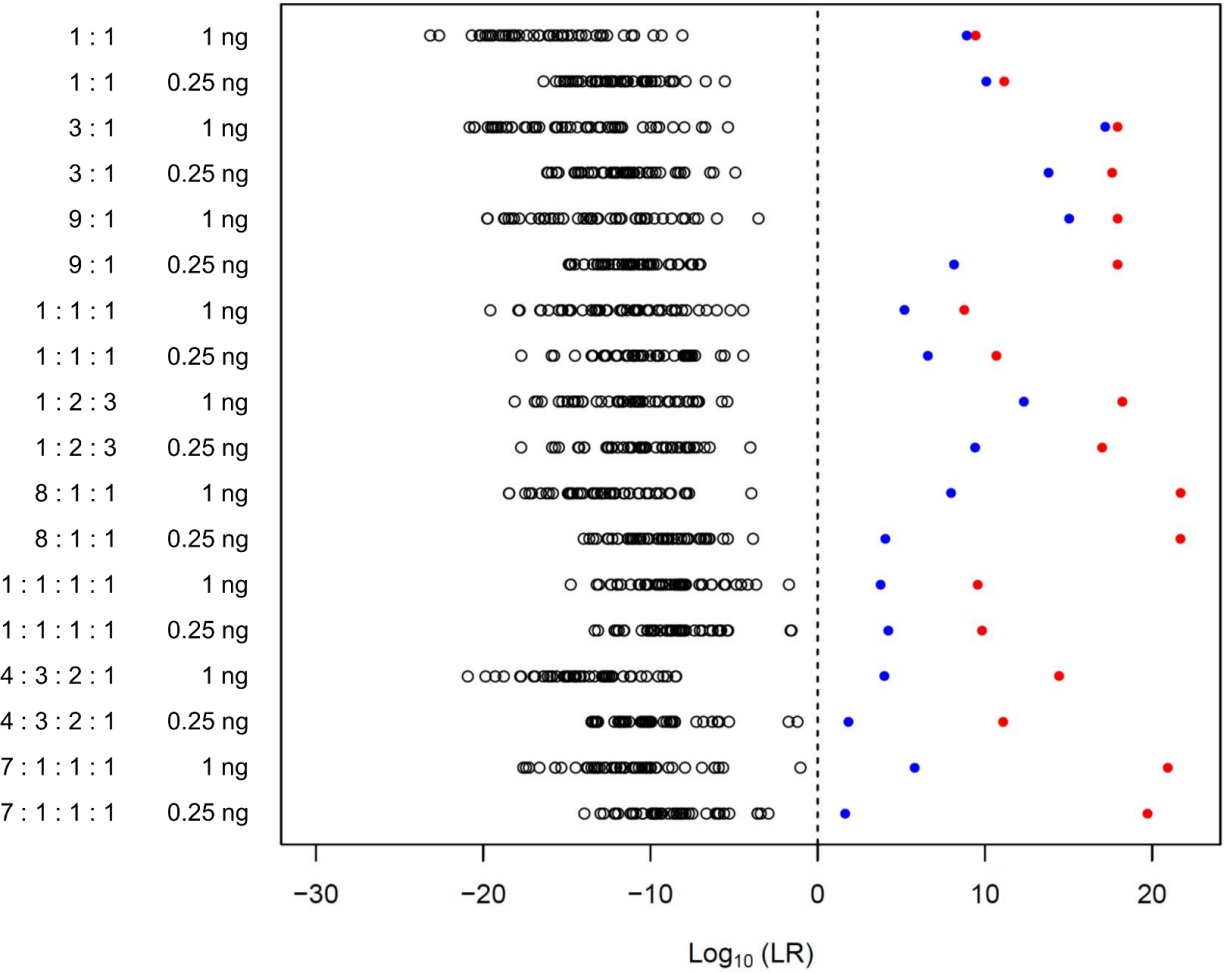
LR values in non-contributor testing are shown in white circles. The red and blue plots represent LR values of the major and minor POIs, respectively.

For validating specificity, we calculated the LR values of computationally generated 100 non-contributor genotypes by using 18 mixtures (Fig 4). All the LR values of the non contributors were below one; therefore, there were no Type II errors (i.e., failure to reject a false hypothesis). On the other hand, all the mixtures indicated that the LR values of the true contributor were greater than those of any other non-contributor. These tendencies were also observed in case-type samples of 9:1, 8:1:1, and 7:1:1:1 mixtures with masked/shared alleles, difficulty in distinguishing minor allelic peaks from stutter peaks, and allelic drop-outs of the minor POI. While the LR values of the true minor contributor in 2 four-person mixtures (i.e., 4:3:2:1 and 7:1:1:1 with 0.25 ng DNA) were smaller than 10,000, most LR values of the non-contributors in these mixtures were significantly small (i.e., LR < 1/10,000).

### Reproducibility of LRs

**Fig 5:**
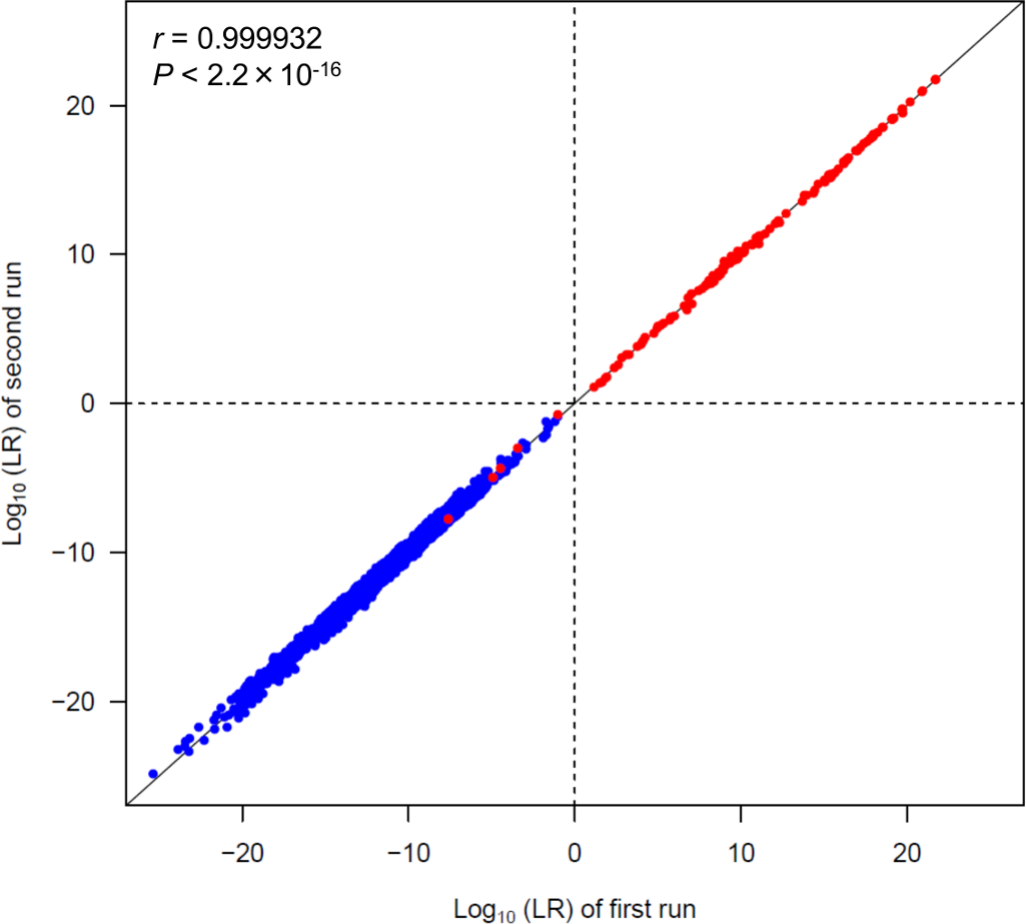
LR values of two runs. The red and blue plots represent the results of true-contributor and non-contributor testing, respectively. The black line represents the *x* = *y* diagonal line and *r* is the correlation coefficient obtained by Pearson’s product-moment correlation test.

For validating precision, we repeated the LR calculation of all 72 mixtures in true-contributor testing and 18 mixtures in non-contributor testing and compared the results of two runs (Fig 5). The plots lie close to the *x* = *y* diagonal line; therefore, there is little variation between each run. The Pearson’s correlation coefficient (*r*) was 0.999932, and the two runs demonstrated a significantly strong correlation (*P* < 2.2 × 10^−16^). In true-contributor testing, the larger LR values were within twice the smaller LR values between two runs in 94.4% mixtures. The ratio of the larger LR to the smaller LR between two runs was at most 3.4 (i.e., LR = 3.33 × 10^9^ and 9.83 × 10^8^ in a 1:1:1 mixture with 1 ng DNA of the POI). In non-contributor testing, the difference of LR values between each run increased slightly. The maximum ratio of the larger LR to the smaller LR between two runs was 10.7 (i.e., LR = 1.49 × 10^−17^ and 1.39 × 10^−18^ in a 4:3:2:1 mixture with 1 ng total DNA). However, in 93.4% non-contributor testing, the larger LR values were within 5 times the smaller LR values. Therefore, there is no discrepancy in the supporting hypothesis between the two runs.

### Accuracy of calculations performed by *Kongoh*

SWGDAM also recommends that studies should assess the accuracy of the calculations performed by the software. We confirmed the accuracy of each calculation performed by the *Kongoh* program, that is, the sampling from the probability distributions of biological parameters, gamma approximation of the expected peak heights, probability density of the observed peak heights given the expected peak heights, calculation of genotype frequencies, and calculation of LR. We also confirmed that the LR values for a major POI in mixtures with completely determined genotype of the POI through the peak height information (e.g., LR of a major POI in 9:1 mixture shown in Fig 4) were the same as those of the reciprocal of the POI genotype frequency.

### LRs of case-type samples

The testing set that includes case-type samples of stutter, masked/shared alleles, and small amounts of DNA has high sensitivity, specificity, and precision as shown in Figs 3–5. We also interpreted the profiles of degradation and inhibition, which were obtained from the PROVEDIt dataset. We investigated 3 two-person mixture profiles with degradation through UV exposure and 3 two-person mixture profiles with inhibition through humic acid. These profiles were 1:4 mixtures of the same two persons, and the total DNA amount was 0.155 ng; however, DNA conditions were different in each. These DNA samples were already genotyped using the Identifiler Plus kit. The DNA damage methods and calculation results of these profiles are shown in Table 1.

**Table 1:** DNA damage method and calculation results of degradation and inhibition profiles.

DNA damage method	Estimated *d* value^a^	LR (minor POI)	LR (major POI)
UV 15 min	−0.0025	2.55 × 10^7^	3.44 × 10^19^
UV 60 min	−0.005	1.62 × 10^3^	2.59 × 10^14^
UV 105 min	−0.0075	1.27 × 10^5^	2.16 × 10^19^
Humic acid 15 µl	0	1.86 × 10^11^	2.24 × 10^22^
Humic acid 22 µl	0	3.62 × 10^6^	1.65 × 10^22^
Humic acid 35 μl	0	4.71 × 10^8^	2.16 × 10^22^

^a^ Estimated ***d*** values in each mixture were the same in both *H*_*p*_ and *H*_*d*_.

In samples with UV exposure, the LR values of the true major POI were strongly supportive of the contribution even in the samples with highly damaged DNA (Table 1). The LR values of the true minor POI were comparatively smaller than those of the major POI but greater than 100,000, except for the sample with 60 min UV exposure. Bright et al. showed that the peak height exponentially decreases with increasing molecular weight of the PCR fragment [28]. We confirmed 9–10 drop-outs of the minor POI alleles; these tended to be observed at loci with higher molecular weight alleles. DNA degradation parameters (***d***) reflect the degree of degradation and tend to be smaller with increasing DNA damage, as explained in S1 Appendix. The estimated ***d*** values demonstrated negative correlation with UV exposure times in both *H*_*p*_ and *H*_*d*_ (Table 1). Therefore, *Kongoh* was able to deal with peak heights appropriately in degraded samples. Loci with lower molecular weight alleles were effective for elucidating a POI’s contribution.

In samples with inhibition through humic acid, the LR values of the true POI were strongly supportive of the contribution, even in the case of 2–7 drop-outs of the minor contributor. Unlike samples with UV exposure, peak heights did not correlate with molecular weights because inhibitors may act at each locus in the same manner regardless of the molecular weights. Therefore, the DNA degradation parameters were estimated as zero (i.e., no degradation).

### Comparison of obtained LRs with those of other models

**Fig 6:**
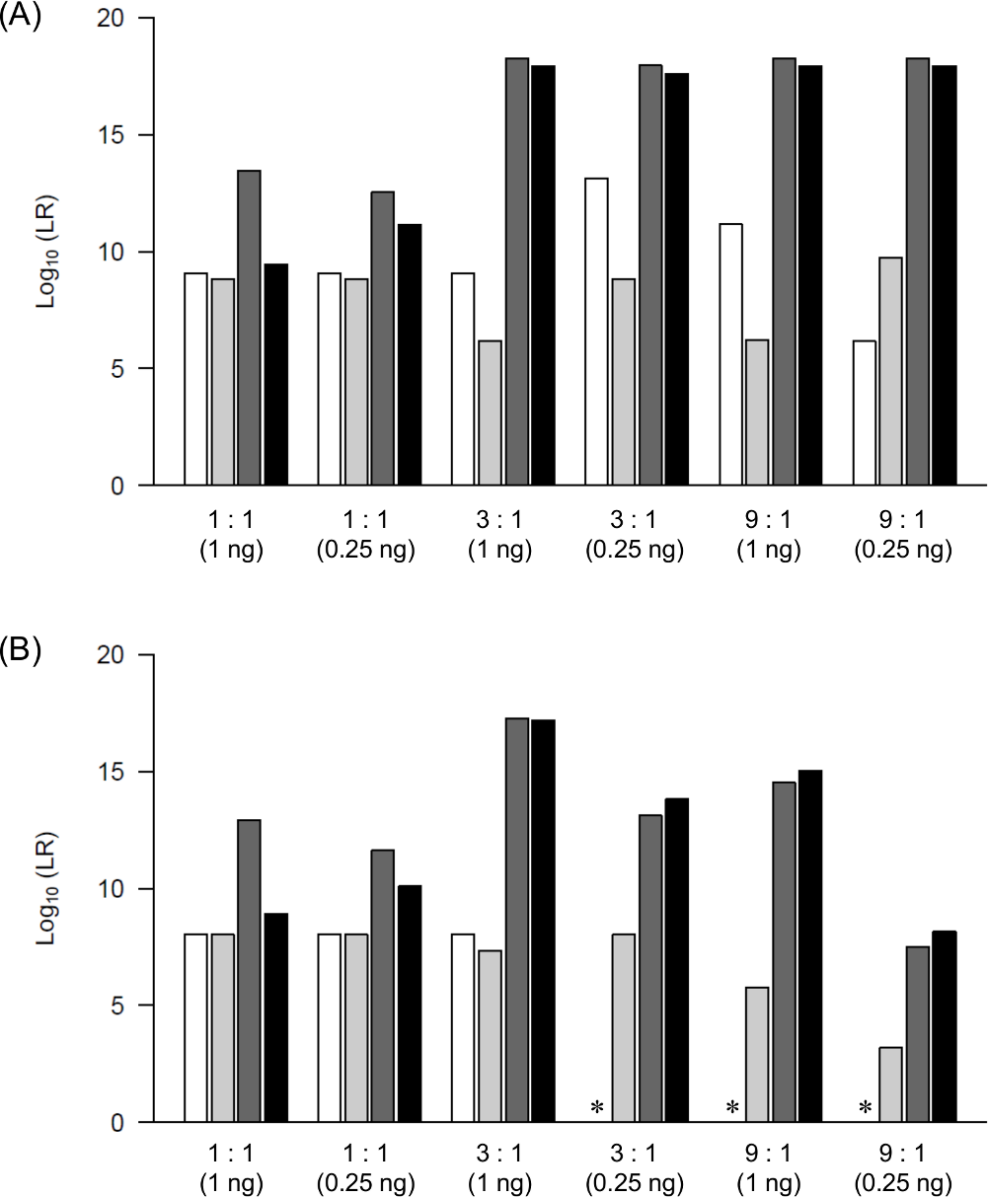
LR values of four models in two-person mixtures. White, light grey, dark grey, and black bars indicate the LR values of binary model, *LRmix Studio*, *EuroForMix*, and *Kongoh*, respectively. (A) LR values of major POI. (B) LR values of minor POI. The asterisks indicate that the LR of binary is zero.

**Fig 7:**
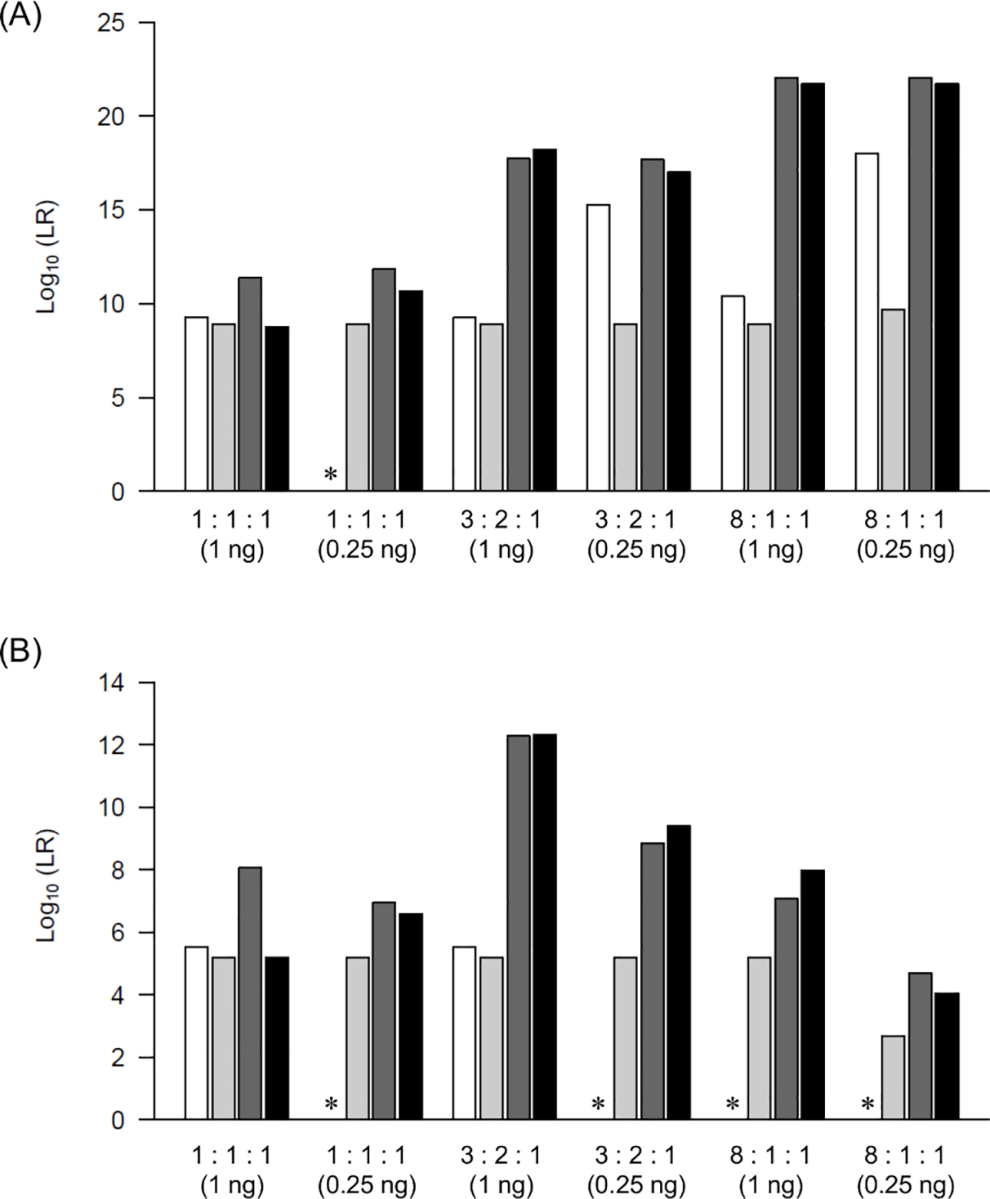
LR values of four models in three-person mixtures. White, light grey, dark grey, and black bars indicate the LR values of binary model, *LRmix Studio*, *EuroForMix*, and *Kongoh*, respectively. (A) LR values of major POI. (B) LR values of minor POI. The asterisks indicate that the LR of binary is zero.

**Fig 8:**
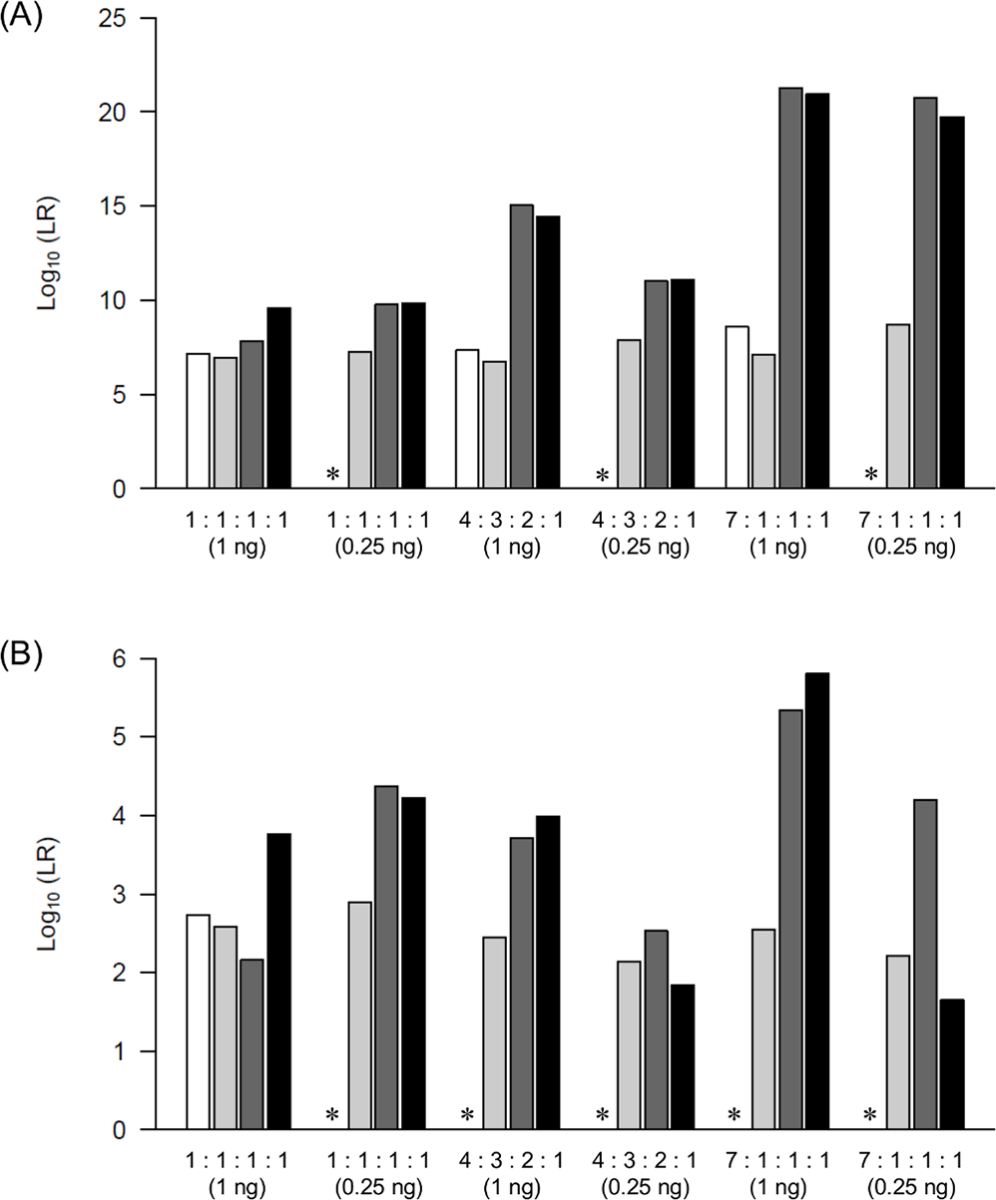
LR values of four models in four-person mixtures. White, light grey, dark grey, and black bars indicate the LR values of binary model, *LRmix Studio*, *EuroForMix*, and *Kongoh*, respectively. (A) LR values of the major POI. (B) LR values of the minor POI. The asterisks indicate that the LR of binary is zero.

We compared the LR values of *Kongoh* with those of a binary model, a qualitative continuous model (*LRmix Studio*), and another quantitative continuous model (*EuroForMix*) to confirm the differences or similarities among the results. The LR values of true-contributor testing in the quantitative continuous model are expected to be greater than those in the binary and qualitative continuous models because peak height information is used in the quantitative continuous model. Both *Kongoh* and *EuroForMix* are based on the quantitative continuous model; therefore, the LR values are expected to be similar. By using 18 mixtures, *Kongoh* generally tended to generate higher LR values than the binary and *LRmix Studio* models, especially in mixtures with different DNA amounts among contributors (Figs 6–8). In mixtures containing the same DNA amounts from all contributors (i.e., 1:1, 1:1:1, and 1:1:1:1), the peak heights had little influence on the increasing of the LR values in *Kongoh* from those in the binary and *LRmix Studio* because similar weights were obtained in each genotype combination without allelic imbalance. Although the LR values of the minor POI in the binary model were mostly zero owing to some of their alleles dropping out (i.e., less than 150 RFU for the analytical threshold in the binary model), high LR values were obtained using *Kongoh*. In *LRmix Studio*, LR = 0 was avoided in mixtures with small DNA amount of POI by using the drop-out probability (Pr(D)). However, strongly supportive LR values (i.e., LR > 10,000) were not obtained in some minor contributors (e.g., 9:1, 3:2:1, or 8:1:1 with 0.25 ng DNA). *Kongoh* generated high LR values in two- and three-person mixtures with small amounts of DNA.

The LR values of *Kongoh* tended to be similar to those of *EuroForMix* even in four-person mixtures; therefore, *Kongoh* could deal with peak heights appropriately based on the quantitative continuous model. However, some LR values of the minor POIs in four-person mixtures were less than 10,000 in both *Kongoh* and *EuroForMix*. In addition, LR variation was observed in minor POI of 7:1:1:1 mixtures with 0.25 ng DNA and with three drop-out alleles of the POI (Fig 8B). This may be attributed to the difference in the computational principle of estimating peak height variances between *Kongoh* and *EuroForMix* especially in small amounts of DNA samples with drop-outs. *Kongoh* estimates peak height distributions by using the Monte Carlo simulation based on experimental data to consider allele- or locus-specific effects. In *EuroForMix*, the expected peak heights are modeled broadly by using gamma distribution, thus providing a versatile method regardless of allele- or locus-specific effects.

### Accuracy of the number of contributors

We also investigated the accuracy of the number of contributors by comparing the likelihoods of 1–4 unknown contributors. The number with the maximum likelihood was determined as the number of contributors. For determining the number of contributors, *Kongoh* demonstrated higher accuracy than any other model (Table 2). Using the binary model, the number of contributors was correctly determined in only 8 out of 18 mixtures (Table 2). In *LRmix Studio* and *EuroForMix*, we may have slight confidence in the determined number because the difference between the maximum likelihood and the second-best likelihood was within 5 times in all mixtures (S1 Table and S2 Table).

**Table 2:** Rate of correctly determined number of contributors in four models.

Mixture	Binary	*LRmix Studio*	*EuroForMix*	*Kongoh*
two-person	5/6	5/6	5/6	6/6
three-person	3/6	0/6	3/6	6/6
four-person	0/6	5/6	3/6	2/6

In *Kongoh*, the number of contributors was correctly determined in all two- and three-person mixtures. The maximum likelihoods were more than 30 times larger than the second-best likelihoods in all mixtures (S2 Table). Four incorrect estimates were observed in four-person mixtures. In particular, in the 7:1:1:1 mixture with 0.25 ng DNA, determining the number of contributors was difficult owing to 14 drop-out alleles and allelic peaks to be distinguished from stutter peaks. In addition, there was slight difference between the maximum likelihood and the second-best likelihood in four-person mixtures even though the number was correctly estimated (S2 Table). Therefore, it is difficult to support the estimated number of contributors strongly in four-person mixtures.

In the total testing set without degradation or inhibition, the number of contributors was correctly determined in 25 out of 27 two-person mixtures, 22 out of 27 three-person mixtures, and 7 out of 18 four-person mixtures using *Kongoh*. While the estimation was especially difficult in four-person mixtures, estimation of the number of contributors in mixtures with 1 ng DNA could be done with high accuracy (i.e., eight out of nine two-person mixtures, eight out of nine three-person mixtures, and four out of six four-person mixtures). In all two-person mixtures with degradation or inhibition, the number of contributors was correctly determined as two. Three- and four-person mixtures with degradation or inhibition will be investigated in future work.

### Other remarks

When we calculated the LR of 72 mixtures by hypothesizing 1–4 contributors, the runtime of analyzing one mixture was approximately 10 h on a standard desktop computer. When we hypothesized 1–3 contributors, the runtime was significantly reduced to only a few minutes. The mixture ratios in both *H*_*p*_ and *H*_*d*_ were nearly exactly estimated for all mixtures including the degraded samples except for those when the number of contributors was incorrectly estimated. The estimated mixture ratios in *H*_*d*_ are shown in S3 Table for mixtures used for the comparative studies and S4 Table for mixtures with degradation or inhibition. The degradation parameters of all the mixtures without degradation were also correctly estimated as zero (i.e., no degradation).

## Discussion

We developed a new open-source software *Kongoh* based on a quantitative continuous model for DNA evidence interpretation. The software automatically calculates the LR values by the ratio of the maximum likelihood in a prosecution hypothesis and in defense hypothesis. Therefore, it does not need to determine the number of contributors manually. However, the number of contributors is generally specified as the same value in both hypotheses prior to analysis. Presciuttini and Egeland regarding hypotheses states that if we are interested in whether the POI is a contributor or not, other parameters such as the number of contributors need to be the same in both prosecution and defense hypothesis [29]. The most probable number of contributors estimated by *Kongoh* in both hypotheses may be different sometimes, but Gill et al. mentioned that there is no reason for the numbers to be the same under alternative hypotheses [30]. *Kongoh* outputs all the likelihoods of 1–4 contributors in both hypotheses as well as the LR value using their maximum likelihoods. Therefore, users can select the likelihoods for any number of contributors to calculate LR.

In the current version of *Kongoh*, we need to prepare profiles typed by the Identifiler Plus kit and analyzed by the ABI 3130xl Genetic Analyzer because *Kongoh* incorporates allele- or locus-specific effects of the typing condition by using experimental data. If we have the experimental data of each parameter from other typing kits or sequencers, we will interpret profiles typed by other systems using *Kongoh*. However, the gamma model (e.g., *EuroForMix*) provides a versatile method by modelling the expected peak heights broadly, regardless of the allele- or locus-specific effects. In this study, *Kongoh* demonstrated higher accuracy of the estimated number of contributors than *EuroForMix* due to incorporating the allele- or locus-specific effects of the system. Therefore, the likelihood values expect to be calculated more rigorously by considering the allele- or locus-specific effects than without these effects.

We validated *Kongoh* by referring to the SWGDAM guidelines, and the LR values generated by *Kongoh* demonstrated high sensitivity, specificity, and precision even in case-type samples. *Kongoh* generated larger LR values than binary and qualitative continuous models, even when some alleles of minor contributors were dropped out. We then confirmed similarities in the LR values obtained by *Kongoh* and other software of the quantitative continuous model (*EuroForMix*). The estimated DNA degradation parameters demonstrated correlation with UV exposure times. Therefore, *Kongoh* was found to deal with peak heights appropriately based on the quantitative continuous model. Furthermore, *Kongoh* achieved higher accuracy of the estimated number of contributors than any other models. Therefore, *Kongoh* is useful in accurate interpretation of DNA evidence such as mixtures and small amounts or degraded DNA samples.

However, some mixtures generated small LR values in true-contributor testing, especially in four-person mixtures with small amount of DNA, many masked/shared alleles, and many allelic drop-outs. In addition, determining the number of contributors was especially difficult in four-person mixtures because there was little difference between the likelihood values of each hypothesized contributor. Therefore, there is a limitation on the number of contributors being more than three in the current Identifiler Plus system. We also have to determine the limitation in DNA with degradation or with PCR inhibition by using three- and four-person mixtures. Additionally, we should calculate LR values by considering the analysis of replicates in the same sample after DNA extraction. Allelic drop-out and heterozygote imbalance stochastically occur in each PCR amplification; therefore, it is common to perform multiple replicates of genotyping to help assess these stochastic effects [4, 31].

*Kongoh* is useful for accurate interpretation of DNA evidence, such as mixtures and small amounts of DNA samples. In future, we intend to apply *Kongoh* to newer STR typing kits with high sensitivity by considering the effect of other artifacts such as forward stutters and -2 backward stutters.

## Supporting information

S1 Appendix Detailed explanation of the calculation principle of the software. (PDF)

S1 Table Likelihoods of 1–4 unknown contributors in binary model and *LRmix Studio*. Boldface denotes the likelihood of the estimated number.
(PDF)

S2 Table Likelihoods of 1–4 unknown contributors in *EuroForMix* and *Kongoh*. Boldface denotes the likelihood of the estimated number.
(PDF)

S3 Table Estimated mixture ratio in samples without DNA damage. (PDF)

S4 Table Estimated mixture ratio in degraded or inhibited 1:4 mixtures with 0.155 ng. (PDF)

S1 Fig An example of a profile analyzed by GeneMapper^TM^ Software. The profile is a 4-person mixture with 1 ng DNA and the mixture ratio is 7:1:1:1. The mixture could be considered as two-person contribution because of many masked/shared alleles.
(PDF)
