# Supplementary material for: Development and validation of open-source software for DNA mixture interpretation based on a quantitative continuous model
Source: PLoS One. 2017 Nov 17;12(11):e0188183. doi: 10.1371/journal.pone.0188183 (PMC5693437; doi:10.1371/journal.pone.0188183)
Supplement: S1 Appendix — (PDF) [file pone.0188183.s001.pdf]

S1 Appendix

We provide a detailed explanation about the likelihood ratio (LR) calculation used in *Kongoh*. In Appendix A, we present a method for determining genotype combinations ($G_{l,i}$) of all contributors in locus l ($i = 1, 2, \dots, I$). In Appendix B, we describe five biological parameters in detail, the mixture ratio (MR_n), DNA degradation (d), locus-specific amplification efficiency (AE_l), heterozygote balance (Hb_{al}), and stutter ratio (SR_{al}), used for calculating the expected peak heights. In Appendix C, we propose a model for the expected peak heights including Monte Carlo simulation of some biological parameters and approximation of gamma distribution. In Appendix D, we explain the method for calculating weight values ($w_{l,i}$) by comparing the observed peak heights with the expected peak heights. In Appendix E, we explain the calculation of the likelihoods in prosecution hypothesis (H_p) and defense hypothesis (H_d).

Appendix A. Determination of genotype combinations

All possible genotype combinations should be considered to calculate the LR values in each locus ($G_{l,i}$ in H_p and $G_{l,i'}$ in H_d). The number of genotype combinations (i.e., I in H_p and I' in H_d) increases with the number of contributors (N). If x alleles are detected in a locus, the number of possible genotypes in a contributor is $x(x+1)/2$. In addition, there is a possibility of stutter and drop-out alleles other than the observed alleles.

To reduce the computational time, *Kongoh* removes some unrealistic genotype combinations according to the following two conditions:

(i) If a stutter ratio is greater than one, the stutter position's peak cannot possibly be derived from only the stutter product.

(ii) If a stutter position's peak is greater than 1,000 RFU, the peak cannot possibly be derived from only the stutter product.

For example, in the profile shown in Fig 1, peak 9 is not composed of only the stutter product because the height of peak 9 is greater than that of peak 10 (this case

corresponds to condition (i)). Peak 11 is not composed of only the stutter product because the height of peak 11 is greater than 1,000 RFU (this case corresponds to condition (ii)). $G_{l,i}$ and $G_{l,i'}$ are obtained after reducing the number of genotype combinations.

In addition, any allele except for the observed peaks may be dropped out. Although the expected peak heights of drop-out alleles are strictly different in each allele, we lump all alleles other than observed peaks together as “Q” for computational convenience [1]. The expected peak height of Q is considered as that of the most frequent allele in Q. The allele frequency of Q is the sum of the frequencies of all alleles other than the observed peaks.

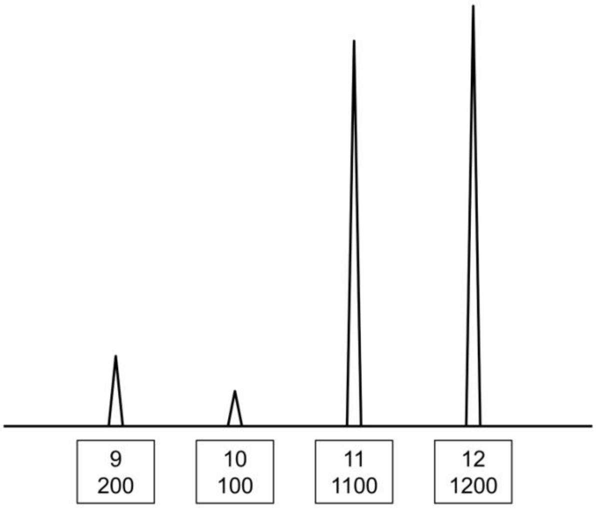

Fig 1. Example of the crime stain profile in a single locus

Appendix B. Parameters

In this section, we describe five biological parameters in detail: mixture ratio (MR_n), DNA degradation (d), locus-specific amplification efficiency (AE_l), heterozygote balance (Hb_{al}), and stutter ratio (SR_{al}) to calculate the expected peak heights. The MR_n and d values in a crime stain profile (CSP) cannot be estimated experimentally because these values differ for each case; therefore, we discretize the two parameters and determine by maximum likelihood estimation (MLE). To perform Monte Carlo simulation, the probability distributions of AE_l, Hb_{al}, and SR_{al} were estimated experimentally by using single-source DNA profiles typed by the AmpFℓSTR® Identifiler® Plus PCR Amplification Kit (Thermo Fisher Scientific, Waltham, MA). Experimental DNA samples were amplified at 28 cycles and analyzed using an ABI 3130xl Genetic Analyzer (Thermo Fisher Scientific) with no enhancements.

Appendix B.1. Mixture ratio

The mixture ratio (MR_n) is the DNA proportion of contributor n ($n = 1, 2, \ldots, N$) in a mixture. In *Kongoh*, two conditions are set for MR_n:

$\sum_n MR_n = 1$,

$0 < MR_1 \leq MR_2 \leq \cdots \leq MR_N < 1 \ (N \geq 2)$.

MR_N is 1 if $N = 1$. MR_n ($N \geq 2$) essentially follows a continuous uniform distribution over the interval (0, 1), but it is computationally convenient to implement a discrete approximation [2]. MR_n is discretized for minor contributors ($n = 1, 2, \ldots, N-1$) into nine values: 0.02, 0.04, 0.06, 0.08, 0.1, 0.2, 0.3, 0.4 and 0.5. MR_n values ($n = 1, 2, \ldots, N-1$) are selected from the nine values to satisfy the two conditions described above, and MR_N is calculated as follows:

$$MR_N = 1 - \sum_{n=1}^{N-1} MR_n.$$

There are many sets of MR_n satisfying the above conditions. In addition to the sets of MR_n calculated by this method, a set of the same amount of DNA in each contributor is added (e.g., $MR_1 = 0.33$, $MR_2 = 0.33$, $MR_3 = 0.34$ for $N = 3$). Therefore, there are 9 sets of MR_n for $N = 2$, 35 sets for $N = 3$, and 98 sets for $N = 4$. The likelihoods (i.e., $f(CSP|H_p)$ and $f(CSP|H_d)$) are calculated under each set of MR_n, and the best set for H_p (MR_{n,H_p}) and H_d (MR_{n,H_d}) is adopted by MLE.

Appendix B.2. DNA degradation

DNA from forensic samples is degraded by some environmental factors. Bright et al. showed that the peak heights exponentially decrease with increase in molecular weight [3]. We denote D_{al} as the relative peak heights of allele a at locus l if the peak height of the mean molecular weight in all the DNA fragments after PCR amplification is one. D_{al} values are calculated using parameter d as follows:

$$D_{al} = \exp(d(m_{al} - \overline{m})),$$

where m_{al} is the molecular weight (base length) of allele a at locus l, and $\overline{m}$ is the mean base length of all DNA fragments after PCR amplification. $\overline{m}$ is not the simple mean of m_{al} of all observed alleles because low-molecular-weight alleles are less affected by degradation than high-molecular-weight alleles. DNA fragments after PCR amplification are mostly derived from low-molecular-weight alleles; therefore, $\overline{m}$ is lower than the simple mean of m_{al}. The difference in the observed peak heights (O_{al}) in each allele is considered for calculating $\overline{m}$ by using the following equation:

$$\bar{m} = \frac{\sum_l \sum_a O_{al} m_{al}}{\sum_l \sum_a O_{al}}.$$

Parameter d reflects the degree of degradation. The degree of degradation should be considered per contributor, but a common d value is used in each contributor for computational convenience. The d value essentially follows continuous uniform distribution over interval $(-\infty, 0]$. When $d = 0$, there is no degradation. For computational convenience, we discretize the d value. The range is defined as $[-0.05, 0]$, which is discretized into 20 intervals of width 0.0025. When $d = -0.05$, DNA has significantly degraded. For example, when an allelic peak height located at the 100 base ($m_{al} = 100$) is 4,000 RFU, the peak height of the other allele located at the 225 base ($m_{al} = 225$, which is approximately the middle detection point in Identifiler Plus system) is expected to be only 8 RFU (i.e., typically less than AT). More than half the alleles should be dropped out when $d = -0.05$; therefore, we assume that the possibility of $d < -0.05$ need not be considered in actual DNA evidence interpretation. We calculate the likelihoods (i.e., $f(CSP|H)$ and $f(CSP|H_d)$) under each d, and the best value for H_p (d_{H_p}) and H_d (d_{H_d}) is adopted by the MLE.

Appendix B.3. Locus-specific amplification efficiency

The locus-specific amplification efficiency (AE_l) is the relative amplification level of each locus. Our experimental data of 234 single-source profiles suggests that AE_l follows a normal distribution in each locus [4]. The variability of AE_l typically increases because of stochastic effects when amplifying low levels of a DNA template. Therefore, the variance of AE_l increases as the amount of DNA template decreases. The truncated ($AE_l \geq 0$) normal distribution is used for generating AE_l in Monte Carlo simulation.

Appendix B.4. Heterozygote balance

Heterozygote balance (Hb_{al}) is the ratio of two heterozygote allelic peaks. Our experimental data suggests that Hb_{al} follows a log-normal distribution in each locus [4]. The variability of $\log_e(Hb_{al})$ increases because of stochastic effects when amplifying low levels of a DNA template. Therefore, the variance of $\log_e(Hb_{al})$ increases as the amount of DNA template decreases. The log-normal distribution is used for generating Hb_{al} in Monte Carlo simulation.

Appendix B.5. Stutter ratio

The stutter ratio (SR_{al}) is the ratio of the stutter peak height and allelic peak height. Our experimental data suggests that a simple log-normal distribution model proposed by Bright et al [5] fit the variability of SR_{al} in all loci except for D8S1179, D21S11 and D2S1338. For D21S11, we develop a new distribution model in which distinct log-normal distributions between complete and incomplete repeat units are used (a separate log-normal distribution model (*sLN*)) [6]. For D8S1179 and D2S1338, we develop a two-component log-normal mixture model (*mLN*) that explains the two types of repeat structures appearing within the same number of allele repeats [6]. We considered the variability of SR_{al} arising from stochastic effects when amplifying low levels of a DNA template by changing the variance of SR_{al} based on the level of a DNA template. Appropriate distributions in each locus are used for generating SR_{al} in Monte Carlo simulation.

Appendix C. Modeling expected peak heights

Appendix C.1. Generating expected peak heights by Monte Carlo simulation

To calculate the weight values ($w_{l,i}$ or $w_{l,i'}$), the expected peak heights (E_{al}) must be modeled using the five biological parameters. Fig 2. shows the procedure for calculating E_{al} in a hypothesized genotype combination. First, the template amount ($\bar{T}$) of a crime stain profile is determined as the starting point for calculating the expected peak heights (Step 1 of Fig 2). The $\bar{T}$ value is calculated as follows:

$$\bar{T} = \frac{\sum_{l=1}^{L} T_l}{L},$$

where T_l denotes the sum of all observed allelic and stutter peak heights in locus l ($l = 1, 2, \dots, L$). The T_l value could be regarded as the template amount in locus l, but the PCR amplification efficiency is different for each locus. Thus, we regard $\bar{T}$ (i.e., mean of T_l) as the template amount of a crime stain profile.

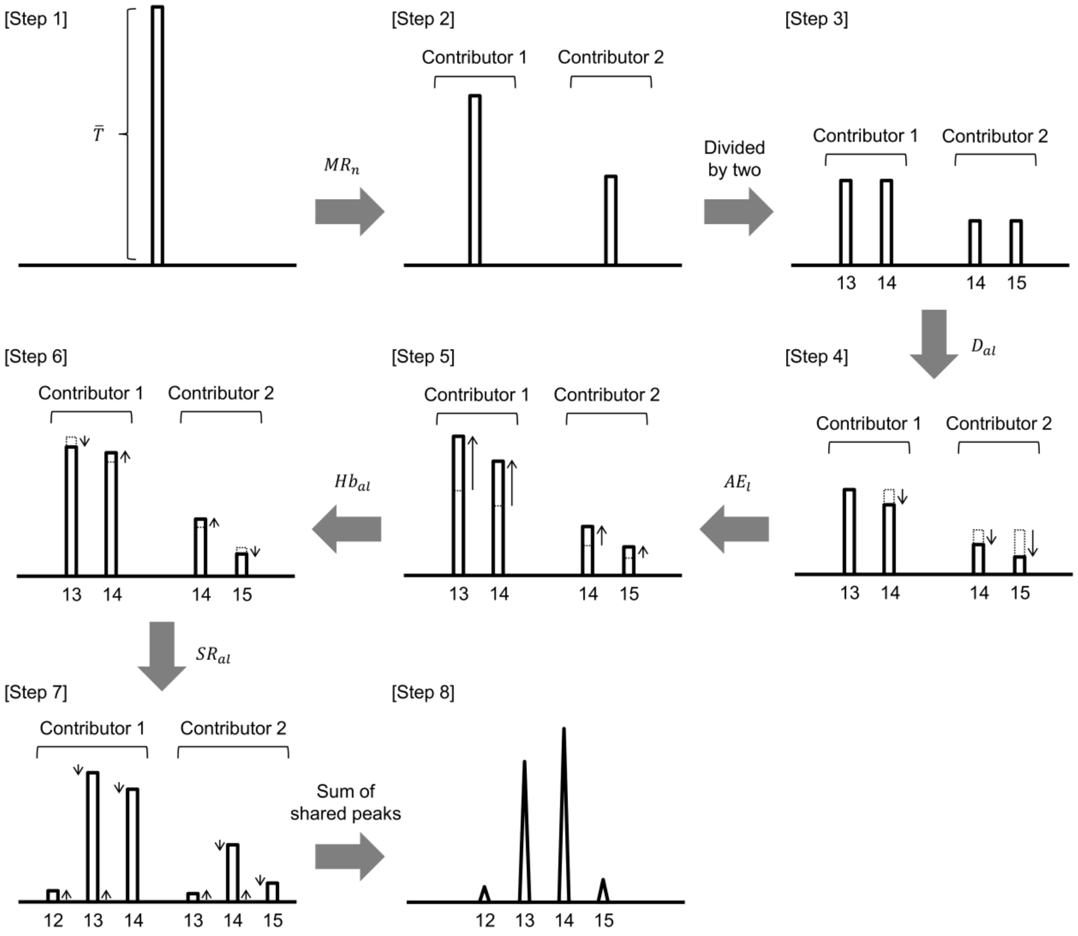

Fig 2. Procedure for calculating E_{al} in a hypothesized genotype combination of two contributors ((13, 14) + (14, 15)). Step 1: Determination of template amount ($\bar{T}$) of a crime stain profile. Step 2: Determination of a set of mixture ratio (MR_n) to divide the template amount into two contributors. Step 3: Division of the template of each contributor into templates of two heterozygote alleles. Step 4: Determination of D_{al} values in each allele. Step 5: Generation of an AE_l value for the locus. Step 6: Generation of Hb_{al} values in each allele. Step 7: Generation of SR_{al} values in each allele. Step 8: Summation of shared peaks in each contributor.

Next, the height of the expected allelic and stutter peaks of contributor n is calculated (E_{aln} and E'_{aln}) using the five biological parameters according to steps 2–7 of the procedure shown in Fig 2. We select a MR_n set in each contributor from discretized values (Step 2), and then the template of each contributor is divided into

templates of two heterozygote alleles (Step 3). Even if a contributor is a homozygote, the template of the contributor is divided into two templates regarded as heterozygote alleles. The expected peak heights of these alleles are summed in Step 8. Next, we select a d value from the discretized values (Step 4) and AE_l (Step 5), Hb_{al} (Step 6), and SR_{al} (Step 7) are randomly selected from the appropriate probability distribution by Monte Carlo simulation. Steps 5–7 are repeated 1,000 times in *Kongoh*, and the selected MR_n and d are fixed during the implementation of Monte Carlo simulation. E_{aln} and E'_{aln} are modeled as follows:

$$E_{aln} = \frac{\bar{T} \cdot MR_n \cdot D_{al} \cdot AE_l}{(1 + Hb_{al}) \cdot (1 + SR_{al})}$$

$$E'_{aln} = \frac{\bar{T} \cdot MR_n \cdot D_{al} \cdot AE_l \cdot SR_{al}}{(1 + Hb_{al}) \cdot (1 + SR_{al})}.$$

As shown in Step 8 of Fig 2, E_{al} is calculated by summing the expected peak heights of allele $\boldsymbol{a}$ (E_{aln}) and expected stutter peak heights of allele $\boldsymbol{a} + 1$ ($E'_{(a+1)ln}$) of all contributors ($n = 1, 2, \dots, N$). Therefore, E_{al} is modeled as follows:

$$E_{al} = \sum_n E_{aln} + \sum_n E'_{(a+1)ln}.$$

If peak $\boldsymbol{a}$ is composed of only allelic product or only stutter product, E_{al} is given by $\sum_n E_{aln}$ or $\sum_n E'_{(a+1)ln}$, respectively.

Appendix C.2. Approximating the variability of expected peak heights using gamma distribution

Allelic peak heights empirically follow gamma distributions [7]. Therefore, the variability of the E_{al} values in each allele or stutter generated by Monte Carlo simulation is approximated by gamma distributions in *Kongoh*. The gamma distribution of E_{al} is expressed as follows:

$$f(E_{al}|k,\theta) = \frac{(E_{al})^{k-1}}{\Gamma(k)\theta^k}\exp\left(-\frac{E_{al}}{\theta}\right).$$

The shape parameter (k) and scale parameter (θ) are determined by MLE. Although our data shows that the SR_{al} values in D8S1179 and D2S1338 follow a bimodal distribution explained by the *mLN* model [6], the distributions of the expected peak heights are not affected significantly by the SR_{al} distribution because of the small

amount of the stutter product.

After approximating the variability of E_{al} using the gamma distribution under $G_{l,i}$, MR_n, and d, the weight values could be calculated by comparing the observed peak heights with the gamma distribution.

Appendix D. Calculating weight values in each genotype combination

The weight ($w_{l,i}$) values are calculated by comparing the observed peak heights (O_{al}) with E_{al} under $G_{l,i}$, selected set of MR_n, and selected d value. If O_{al} values are well fitted to E_{al} values, $w_{l,i}$ is comparatively high. In contrast, if there is a large difference between O_{al} and E_{al}, $w_{l,i}$ is comparatively low. To investigate the difference in locus l, O_{al} $(a = 1, 2, \dots, A)$ is applied to each appropriate gamma distribution of E_{al}, and we obtain the probability density of observing O_{al} given $G_{l,i}$, MR_n, and d. The probability density (i.e., the weight) is calculated by multiplying the y-axis values of all O_{al} values in each distribution. The equation is as follows:

$$w_{l,i} = f\left(CSP_l \middle| G_{l,i}, MR_n, d\right) = f\left(O_{1l}, \dots, O_{Al} \middle| G_{l,i}, MR_n, d\right) = \prod_a f(O_{al} | G_{l,i}, MR_n, d).$$

When the expected peak is not observed (the hypothesized allele is less than AT), we consider that the possible O_{al} values are integers between 1 to AT-1, each of which are assumed to be equally probable. We do not assume that the O_{al} for the drop-out peak is zero because the range of the expected peak heights based on the gamma distribution is greater than zero. For the drop-out peak, $f(O_{al}|G_{l,i}, MR_n, d)$ is calculated as follows:

$$f\left(O_{al}\middle|G_{l,i}, MR_n, d\right) = \frac{\sum_{Z=1}^{\mathrm{AT}-1} f\left(Z\middle|G_{l,i}, MR_n, d\right)}{\mathrm{AT}-1}.$$

Appendix E. Calculating likelihoods

After calculating the $w_{l,i}$ values (i.e., $f\left(CSP_l\middle|G_{l,i}, MR_n, d\right)$) in each $G_{l,i}$, MR_n, and d of all loci, a likelihood value under a hypothesis (H) can be calculated as follows:

$$f(CSP|H) = \prod_l \sum_i f\left(CSP_l\middle|G_{l,i}, MR_n, d\right) \Pr(G_{l,i}|H).$$

The likelihoods of each number of contributors, the set of MR_n, and d are calculated. The best likelihood is determined by MLE in both H_p and H_d. We recommend that the

ratio of the maximum likelihood in H_p and that in H_d is the LR of CSP, which is calculated as follows:

$$\mathrm{LR} = \frac{\prod_l \sum_i f\left(CSP_l \middle| G_{l,i}, MR_{n,H_p}, d_{H_p}\right) \Pr(G_{l,i}|H_p)}{\prod_l \sum_{i'} f\left(CSP_l \middle| G_{l,i'}, MR_{n,H_d}, d_{H_d}\right) \Pr(G_{l,i'}|H_d)}$$

References

1. Balding DJ, Buckleton J. Interpreting low template DNA profiles. Forensic Sci Int Genet. 2009; 4: 1-10.

2. Puch-Solis R, Rodgers L, Mazumder A, Pope S, Evett I, Curran J, et al. Evaluating forensic DNA profiles using peak heights, allowing for multiple donors, allelic dropout and stutters. Forensic Sci Int Genet. 2013; 7: 555-63.

3. Bright J-A, Taylor D, Curran J, Buckleton JS. Degradation of forensic DNA profiles. Aust J Forensic Sci. 2013; 45: 445-9.

4. Manabe S, Hamano Y, Kawai C, Morimoto C, Tamaki K. Development of new peak-height models for a continuous method of mixture interpretation. Forensic Sci Int Genet Suppl Ser. 2015; 5: e104-e6.

5. Bright J-A, Taylor D, Curran JM, Buckleton JS. Developing allelic and stutter

peak height models for a continuous method of DNA interpretation. Forensic Sci Int Genet. 2013; 7: 296-304.

6. Manabe S, Hamano Y, Morimoto C, Kawai C, Fujimoto S, Tamaki K. New stutter ratio distribution for DNA mixture interpretation based on a continuous model. Leg Med (Tokyo). 2016; 19: 16-21.

7. Cowell RG, Lauritzen SL, Mortera J. A gamma model for DNA mixture analyses. Bayesian Anal. 2007; 2: 333-48.
