# Supplementary material for: Development and validation of open-source software for DNA mixture interpretation based on a quantitative continuous model
Source: PLoS One. 2017 Nov 17;12(11):e0188183. doi: 10.1371/journal.pone.0188183 (PMC5693437; doi:10.1371/journal.pone.0188183)
Supplement: S1 Table — Boldface denotes the likelihood of the estimated number. (PDF) [file pone.0188183.s002.pdf]

S1 Table

Mixture Ratio	DNA amount (ng)	Binary				*LRmix Studio*			
		one-person	two-person	three-person	four-person	one-person	two-person	three-person	four-person
1 : 1	1	0	**7.53×10^{-22}**	1.46×10^{-25}	3.47×10^{-31}	0	**8.09×10^{-22}**	3.67×10^{-22}	3.81×10^{-22}
1 : 1	0.25	0	**7.53×10^{-22}**	1.46×10^{-25}	3.47×10^{-31}	0	**8.09×10^{-22}**	3.67×10^{-22}	3.81×10^{-22}
3 : 1	1	0	**7.53×10^{-22}**	1.46×10^{-25}	3.47×10^{-31}	0	4.89×10^{-22}	6.14×10^{-22}	**8.44×10^{-22}**
3 : 1	0.25	0	**5.39×10^{-25}**	2.46×10^{-34}	1.61×10^{-44}	0	**8.09×10^{-22}**	3.67×10^{-22}	3.81×10^{-22}
9 : 1	1	0	**1.05×10^{-23}**	4.44×10^{-29}	3.94×10^{-36}	0	3.05×10^{-23}	9.83×10^{-23}	**1.79×10^{-22}**
9 : 1	0.25	**2.55×10^{-18}**	8.64×10^{-28}	1.10×10^{-39}	4.42×10^{-52}	0	**4.21×10^{-21}**	3.39×10^{-21}	4.16×10^{-21}
1 : 1 : 1	1	0	0	**3.18×10^{-28}**	2.14×10^{-29}	0	0	3.18×10^{-28}	**6.91×10^{-28}**
1 : 1 : 1	0.25	0	**9.82×10^{-23}**	6.31×10^{-28}	6.01×10^{-35}	0	0	3.18×10^{-28}	**6.91×10^{-28}**
3 : 2 : 1	1	0	0	**3.18×10^{-28}**	2.14×10^{-29}	0	0	3.18×10^{-28}	**6.91×10^{-28}**
3 : 2 : 1	0.25	0	**8.49×10^{-29}**	2.26×10^{-36}	3.64×10^{-45}	0	0	3.18×10^{-28}	**6.91×10^{-28}**
8 : 1 : 1	1	0	0	**4.72×10^{-30}**	3.30×10^{-33}	0	0	3.18×10^{-28}	**6.91×10^{-28}**
8 : 1 : 1	0.25	0	**6.77×10^{-32}**	1.08×10^{-43}	4.20×10^{-56}	0	0	3.01×10^{-27}	**3.74×10^{-27}**
1 : 1 : 1 : 1	1	0	0	**1.95×10^{-27}**	5.44×10^{-28}	0	0	1.95×10^{-27}	**3.17×10^{-27}**
1 : 1 : 1 : 1	0.25	0	0	0	0	0	0	1.83×10^{-27}	**2.47×10^{-27}**
4 : 3 : 2 : 1	1	0	0	**1.11×10^{-27}**	1.02×10^{-28}	0	0	2.30×10^{-28}	**4.62×10^{-28}**
4 : 3 : 2 : 1	0.25	0	0	0	0	0	0	**1.03×10^{-28}**	9.94×10^{-29}
7 : 1 : 1 : 1	1	0	9.93×10^{-26}	**2.87×10^{-25}**	3.24×10^{-28}	0	0	5.79×10^{-29}	**1.32×10^{-28}**
7 : 1 : 1 : 1	0.25	**1.08×10^{-22}**	9.92×10^{-38}	5.23×10^{-54}	1.46×10^{-70}	0	4.77×10^{-25}	3.93×10^{-25}	**4.84×10^{-25}**
