# Supplementary material for: Development and validation of open-source software for DNA mixture interpretation based on a quantitative continuous model
Source: PLoS One. 2017 Nov 17;12(11):e0188183. doi: 10.1371/journal.pone.0188183 (PMC5693437; doi:10.1371/journal.pone.0188183)
Supplement: S2 Table — Boldface denotes the likelihood of the estimated number. (PDF) [file pone.0188183.s003.pdf]

S2 Table

Mixture Ratio	DNA amount (ng)	*EuroForMix*				*Kongoh*			
		one-person	two-person	three-person	four-person	one-person	two-person	three-person	four-person
1 : 1	1	0	**3.15×10^{-218}**	3.15×10^{-218}	3.15×10^{-218}	0	**1.04×10^{-198}**	1.06×10^{-204}	1.44×10^{-211}
1 : 1	0.25	0	**9.08×10^{-162}**	9.08×10^{-162}	9.08×10^{-162}	0	**3.02×10^{-154}**	9.85×10^{-160}	6.40×10^{-165}
3 : 1	1	0	**1.63×10^{-217}**	1.63×10^{-217}	1.63×10^{-217}	0	**4.41×10^{-204}**	1.95×10^{-207}	2.97×10^{-213}
3 : 1	0.25	0	**4.07×10^{-160}**	4.07×10^{-160}	4.07×10^{-160}	0	**2.04×10^{-153}**	5.20×10^{-157}	4.86×10^{-162}
9 : 1	1	0	4.39×10^{-201}	**4.89×10^{-201}**	4.89×10^{-201}	0	**1.56×10^{-190}**	4.51×10^{-192}	3.06×10^{-197}
9 : 1	0.25	0	**4.26×10^{-145}**	4.26×10^{-145}	4.26×10^{-145}	0	**3.15×10^{-139}**	6.63×10^{-144}	3.29×10^{-148}
1 : 1 : 1	1	0	1.72×10^{-284}	4.07×10^{-266}	**4.24×10^{-266}**	0	0	**2.39×10^{-246}**	4.01×10^{-249}
1 : 1 : 1	0.25	0	5.31×10^{-183}	2.31×10^{-176}	**2.66×10^{-176}**	0	0	**1.29×10^{-171}**	1.96×10^{-173}
3 : 2 : 1	1	0	4.45×10^{-278}	**1.60×10^{-271}**	1.60×10^{-271}	0	0	**1.96×10^{-255}**	9.64×10^{-259}
3 : 2 : 1	0.25	0	1.42×10^{-180}	**2.40×10^{-177}**	2.40×10^{-177}	0	0	**3.87×10^{-174}**	3.30×10^{-176}
8 : 1 : 1	1	0	5.91×10^{-252}	**4.92×10^{-241}**	4.92×10^{-241}	0	0	**2.14×10^{-222}**	8.59×10^{-226}
8 : 1 : 1	0.25	0	3.18×10^{-172}	3.24×10^{-170}	**3.28×10^{-170}**	0	5.41×10^{-190}	**4.63×10^{-168}**	7.66×10^{-172}
1 : 1 : 1 : 1	1	0	0	**7.93×10^{-271}**	7.93×10^{-271}	0	0	**2.58×10^{-244}**	2.91×10^{-245}
1 : 1 : 1 : 1	0.25	0	2.07×10^{-175}	4.50×10^{-169}	**3.38×10^{-168}**	0	0	**6.26×10^{-166}**	5.30×10^{-166}
4 : 3 : 2 : 1	1	0	0	7.77×10^{-268}	**9.64×10^{-268}**	0	0	3.48×10^{-261}	**9.10×10^{-253}**
4 : 3 : 2 : 1	0.25	0	6.74×10^{-171}	**3.06×10^{-162}**	3.06×10^{-162}	0	0	**7.37×10^{-165}**	2.35×10^{-166}
7 : 1 : 1 : 1	1	0	0	1.69×10^{-254}	**3.24×10^{-253}**	0	0	1.47×10^{-236}	**2.55×10^{-234}**
7 : 1 : 1 : 1	0.25	0	2.29×10^{-137}	**3.28×10^{-136}**	3.28×10^{-136}	0	**7.44×10^{-144}**	1.63×10^{-144}	1.15×10^{-148}
