# Supplementary material for: Development and validation of open-source software for DNA mixture interpretation based on a quantitative continuous model
Source: PLoS One. 2017 Nov 17;12(11):e0188183. doi: 10.1371/journal.pone.0188183 (PMC5693437; doi:10.1371/journal.pone.0188183)
Supplement: S3 Table — (PDF) [file pone.0188183.s004.pdf]

S3 Table

DNA amount (ng)	Actual mixture ratio	Estimated mixture ratio in H_d
1	1 : 1	0.6 : 0.4
0.25	1 : 1	0.6 : 0.4
1	3 : 1	0.8 : 0.2
0.25	3 : 1	0.8 : 0.2
1	9 : 1	0.9 : 0.1
0.25	9 : 1	0.9 : 0.1
1	1 : 1 : 1	0.4 : 0.3 : 0.3
0.25	1 : 1 : 1	0.34 : 0.33 : 0.33
1	3 : 2 : 1	0.5 : 0.3 : 0.2
0.25	3 : 2 : 1	0.5 : 0.3 : 0.2
1	8 : 1 : 1	0.8 : 0.1 : 0.1
0.25	8 : 1 : 1	0.8 : 0.1 : 0.1
1	1 : 1 : 1 : 1	0.5 : 0.3 : 0.2
0.25	1 : 1 : 1 : 1	0.4 : 0.3 : 0.3
1	4 : 3 : 2 : 1	0.5 : 0.2 : 0.2 : 0.1
0.25	4 : 3 : 2 : 1	0.4 : 0.3 : 0.3
1	7 : 1 : 1 : 1	0.74 : 0.1 : 0.1 : 0.06
0.25	7 : 1 : 1 : 1	0.8 : 0.2
