# Supplementary material for: Development and validation of open-source software for DNA mixture interpretation based on a quantitative continuous model
Source: PLoS One. 2017 Nov 17;12(11):e0188183. doi: 10.1371/journal.pone.0188183 (PMC5693437; doi:10.1371/journal.pone.0188183)
Supplement: S4 Table — (PDF) [file pone.0188183.s005.pdf]

S4 Table

DNA damage method	Estimated mixture ratio in H_d
UV 15 min	0.1 : 0.9
UV 60 min	0.2 : 0.8
UV 105 min	0.3 : 0.7
Humic acid 15 μl	0.2 : 0.8
Humic acid 22 μl	0.2 : 0.8
Humic acid 35 μl	0.2 : 0.8
