# Supplementary figures and images for: Development and validation of open-source software for DNA mixture interpretation based on a quantitative continuous model
Source: PLoS One. 2017 Nov 17;12(11):e0188183. doi: 10.1371/journal.pone.0188183 (PMC5693437; doi:10.1371/journal.pone.0188183)

S1 Fig

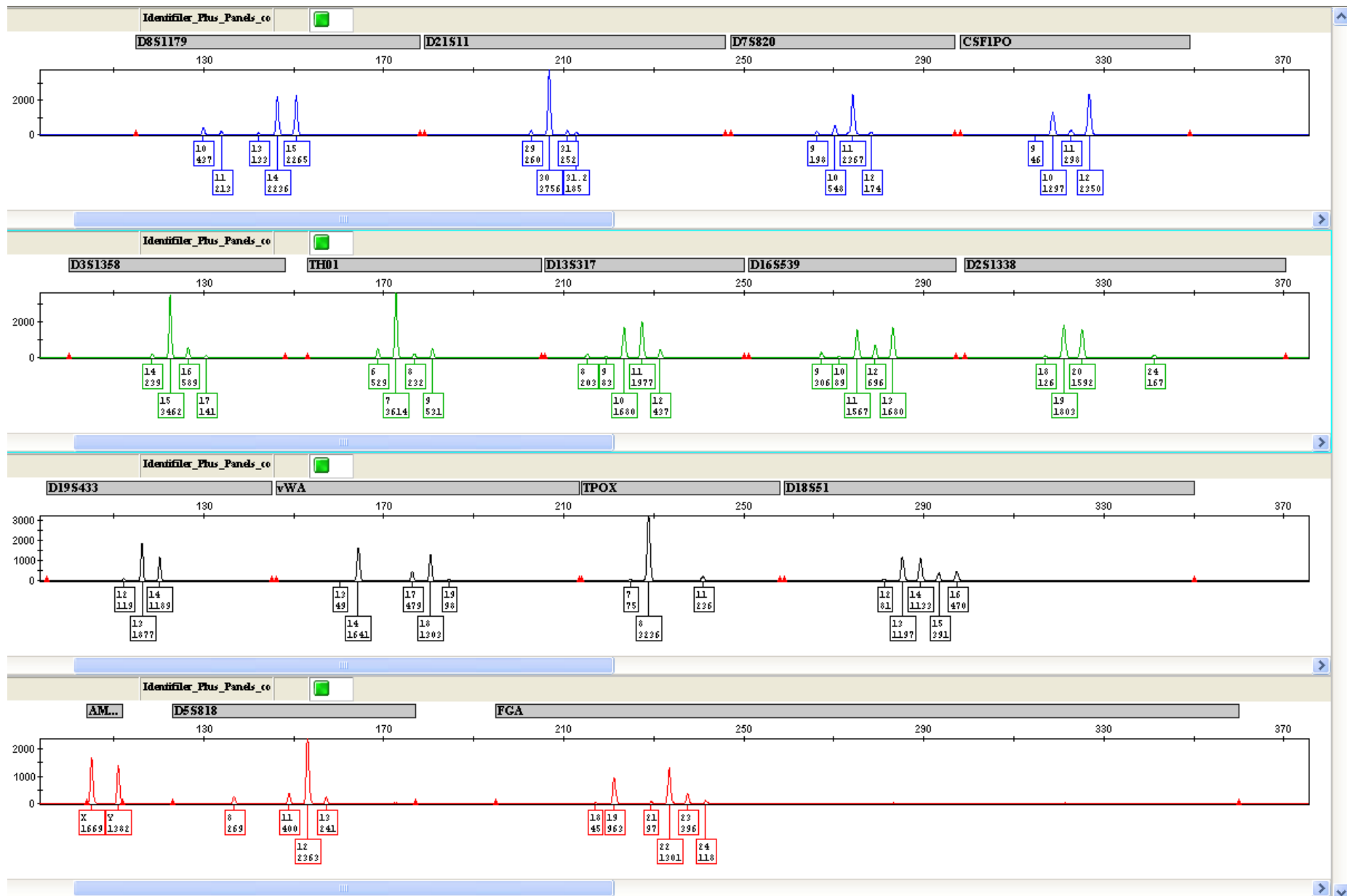

Supplement: S1 Fig — The profile is a 4-person mixture with 1 ng DNA and the mixture ratio is 7:1:1:1. The mixture could be considered as two-person contribution because of many masked/shared alleles. (PDF) [file pone.0188183.s006.pdf]
